# Effect of mobile health interventions in increasing utilization of Maternal and Child Health care services in developing countries: A scoping review

**DOI:** 10.1177/20552076221143236

**Published:** 2022-12-13

**Authors:** Ramachandran Venkataramanan, S.V. Subramanian, Mohannad Alajlani, Theodoros N Arvanitis

**Affiliations:** 1 Institute of Digital Healthcare, 120959 WMG – The University of Warwick, Coventry, USA; 2 Research Division, Karkinos Healthcare, Mumbai, India; 3 Harvard Center for Population & Development Studies, Cambridge, MA, USA; 4 Department of Social and Behavioral Sciences, 1857 Harvard T.H. Chan School of Public Health, Boston, MA, USA

**Keywords:** Public health, mHealth, digital health, behavior change, neonatal, mobile phone

## Abstract

**Background:**

Mobile health (mHealth) technology is being used predominantly in low- and middle-income countries. Developing countries with low level of investment in health infrastructure can augment existing capacity by adopting low-cost affordable technology. The aim of the review was to summarize the available evidence on mHealth interventions that aimed at increasing the utilization of Maternal and Child Health (MCH) care services. Further, this review investigated the barriers which prevent the use of mHealth among both health care workers as well as beneficiaries.

**Methodology:**

A scoping review of literature was undertaken using the five-stage framework developed by Arksey and O’Malley. The articles published between 1990 and 2021 were retrieved from three databases (PubMed, Cochrane Reviews, and Google Scholar) and grey literature for this review. The Preferred Reporting Items for Systematic Reviews and Meta-Analyses extension for Scoping Reviews (PRISMA-ScR) checklist was followed to present the findings.

**Result:**

A total of 573 studies were identified. After removing duplicates, studies not related to mHealth and MCH and publications of systematic reviews and protocols for studies, a total of 28 studies were selected for review. The study design of the research articles which appeared during the search process were mostly observational, cross-sectional, and randomized controlled trials (RCTs). We have classified the studies into four categories based on the outcomes for which the mHealth intervention was implemented: MCH care services, child immunization, nutrition services, and perceptions of stakeholders toward using technology for improving MCH outcomes.

**Conclusion:**

This brief review concludes that mHealth interventions can improve access to MCH services. However, further studies based on large sample size and strong research design are recommended.

## Introduction

The deadline to achieve the Millennium Development Goals (MDGs), which were set following the Millennium Summit of the United Nations in 2000 with an aim to improve the lives of the poor people, came to an end in 2015. Maternal and child mortality continues to remain high across developing countries. The targets which remain a concern for the policymakers are reduction in maternal mortality ratio (MMR) which is defined as the number of maternal deaths per 100,000 live births and utilization of reproductive health services. The fifth MDG aimed at a reduction of 75% in MMR over the period 1990 to 2015. However, globally, MMR fell by 44% during this period which was well short of target.^1^ It was estimated that 295,000 women died in 2017 due to pregnancy and childbirth-related complications and majority of these deaths occurred in low resource settings.^1,2^ The Sustainable Development Goal (SDG) target is to reduce the global MMR to 70 per 100,000 live births by 2030. In addition, ensuring child immunization could save a number of preventable child deaths. However, over the past few years, a drop in coverage of child immunization has been observed.^3^ Multiple outbreaks of measles, diphtheria, and other vaccine preventable diseases were observed recently even before the outbreak of the COVID-19 pandemic.^1^

Primary health care (PHC) services provided during pregnancy and timely vaccination can prevent numerous birth-related complications and lower the risk of premature mortality among children.^1^ Most of the complications which result in these deaths are easily treatable but the access to services remain low. The reason for low utilization of services is low level of public investment, inadequate and crumbling infrastructure, and shortage of skilled human resources, particularly in rural areas of the developing countries.^4,5^

Digital health is emerging as a viable solution to ensure safe pregnancy and improve pregnancy outcomes.^6^ Digital health encompasses many sub-sectors including ehealth, mobile heath (mHealth), telehealth, health information technology, and telemedicine. More specifically, the term digital health is defined as “a broad umbrella term encompassing eHealth (which includes mHealth), as well as emerging areas, such as the use of advanced computing sciences in ‘big data’, genomics and artificial intelligence.”^7^ WHO defines mHealth as “medical and public health practice supported by mobile devices, such as mobile phones, patient monitoring devices, personal digital assistants, and other wireless devices.”^8^

In particular, mHealth technology is being used predominantly in developing countries. As per Labrique et al., 12 uses of mHealth technology can be identified with the most effective being the ability to modify the behavior and to provide information to both supply and demand-side stakeholders.^9^ On the supply side, the community health workers could be trained to collect data, send reminders, and monitor the schedules related with service delivery. Similarly, on the demand side, the pregnant and lactating women can contact the service providers, book appointments, gain timely information, and call out for ambulance services in remote areas.

The effectiveness of information technology in improving coverage as well as quality of health care services is well documented.^10,11^ The greatest benefit is usually derived by regions, which due to structural deficiencies and resource poor settings, have registered low access and utilization. In particular, mHealth interventions have the potential to improve Maternal and Child Health (MCH) outcomes and to reduce the burden of deaths due to easily preventable diseases.^12,13^ By adopting low-cost affordable technology, the existing capacity can be augmented in developing countries.^7^ Augmenting the knowledge base through use of short messaging service (SMS) texts and voice calls can be instrumental in changing the health behavior.^6^

However, there are many challenges to be overcome to successfully implement mHealth interventions. Barboni et al. have categorized the barriers as normative and economic.^14^ Normative barriers arise on account of traditional beliefs, customs, and social norms. Economic barriers are related to infrastructure, cost of handset, and financial needs. The socio-economic inequalities could determine the access to mobile phones and lead to inequalities in health outcomes. Those who have a mobile might be able to leverage its potential when compared to nonmobile owners. For instance, women who have a mobile phone are more likely to avail health care services during pregnancy.^15^ Also, mobile phone interventions can be used to connect those from marginalized groups with the health providers.^16^ Mobile phone ownership in settings which have mHealth intervention is significantly associated with higher utilization of maternal health services.^17,18^

As per our research, four systematic reviews have been conducted on the effectiveness of mHealth for maternal care. Colaci et al. assessed 19 studies to understand the use of mHealth for maternal health in low- and middle-income countries.^19^ Six of these studies highlighted the use of mHealth for provider-to-provider communication and while rest of the 13 studies focused on the use of mHealth for reminders and data collection. Chen et al. analyzed the effectiveness of mHealth for MCH and found that 43% of the 51 randomized controlled trials (RCTs) results yielded inconsistent results.^20^ Lee et al. analyzed the effectiveness of various mHealth interventions. The outcomes considered in their study were maternal and child morbidity and mortality, antenatal care (ANC) visits, immunization, and other process indicators.^5^ Sondaal et al. found that most of the mHealth interventions improved the uptake of maternal health care services however the impact on maternal outcomes was inconsistent.^12^ There is a broad consensus across these studies that impact of mHealth interventions is inconsistent in different settings.

Against this background, the aim was to identify the studies for developing countries which evaluated the success of mHealth interventions in increasing the utilization of MCH care services (particularly immunization) and barriers which prevented the use among both health care workers as well as beneficiaries.

## Data and methods

**Figure 1.**
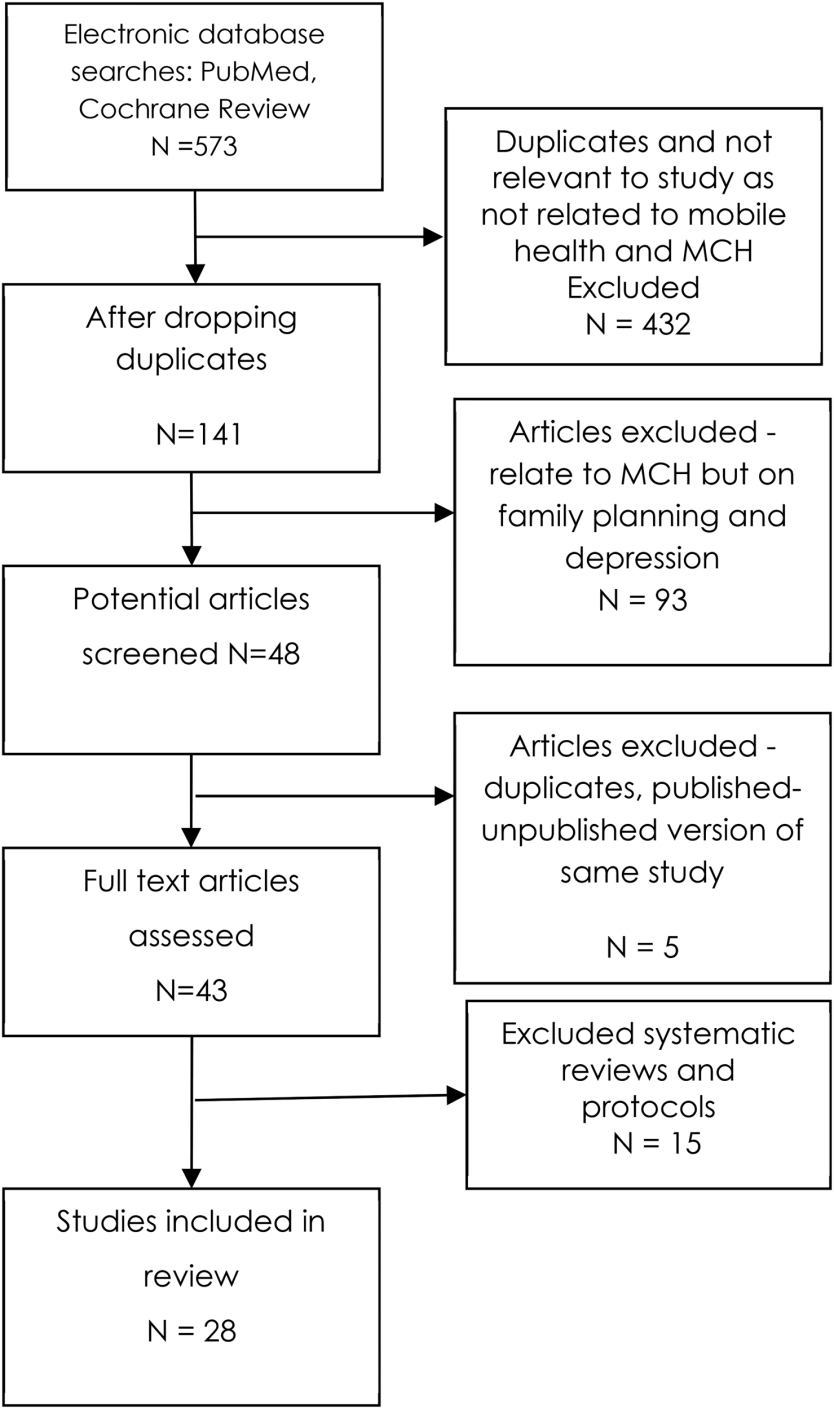
Description of steps followed to shortlist studies based on Preferred Reporting Items for Systematic Reviews and Meta-Analyses extension for Scoping Reviews (PRISMA-ScR) guidelines.

Our scoping review followed the five-stage framework developed by Arksey and O’Malley: (1) identifying the research or review question(s), (2) identifying relevant studies, (3) selecting the studies for review, (4) charting the data from them; and (5) collating, summarizing, and reporting results.^21^ The Preferred Reporting Items for Systematic Reviews and Meta-Analyses extension for Scoping Reviews (PRISMA-ScR) checklist was followed to present the findings (Figure 1).^22^

### Identifying the research question

The research question for this review was to understand the purpose and impact of mHealth interventions which are being used for MCH care. More specifically, the following research questions were investigated: (1) What key components of MCH care services are the focus of the mHealth interventions? (2) What are the key utilities of mobile phones used to deliver the intervention? (3) What are the outcomes in terms of improvement in utilization of MCH care services?

### Eligibility criteria

Studies focusing on the coverage of MCH care services before pregnancy, during pregnancy and post-pregnancy were considered. Search was conducted to identify the impact of mHealth intervention on the following services: ANC, postnatal checkup, breastfeeding, and child immunization. mHealth was defined as “medical and public health practice supported by mobile devices, such as mobile phones, patient monitoring devices, personal digital assistants, and other wireless devices”. In context of this review, we focused on use of voice calls and text messages to exchange information between providers and beneficiaries. Further, only research articles published in English language were considered for the review.

### Information sources

Initially, the search was conducted between June and September 2021 by RV and MA. We considered three online databases which included PubMed, Cochrane Library, and Google Scholar. Article published between 1990 and 2021 were retrieved using these databases.

### Key terms

Search was conducted using a combination of terms including “SMS text”, “immunization coverage”, “vaccination coverage”, “mhealth”, “maternal and child health service utilization”, “developing countries”, and “maternal and child nutrition”.

### Study selection and data charting process

A review of literature was conducted based on articles published between 1990 and 2021. There were very few studies which were published prior to 2010. Two authors (RV and MA) were involved in identifying the studies based on title and abstract, which were retrieved on the basis of certain keywords. The aim was to identify the studies for developing countries which evaluated the success of mhealth interventions in increasing the utilization of MCH care services (particularly immunization) and barriers which prevented the use among both health care workers as well as beneficiaries. The research was considered to be rigorous, if the method to evaluate the health intervention involved RCTs. The search information was extracted by authors (RV and MA) on study design, country, and type of mHealth intervention. As a priori, we had information about the type of mHealth interventions and purpose but the themes for presenting the results were identified after the search.

### Synthesis of results

The studies were categorized based on the outcome being analyzed. The themes were classified based on information related to MCH care services. Data has been presented in tabular form. Information on study design, country, and type of technology and health outcome are presented. The results have been summarized in Table 1.

**Table 1.** Summary of selected studies for review.

S. No.	Author	Study design	Sample size	Country	mHealth intervention	Health indicator	Key findings
1	Kazi et al. 2014^23^	Random sampling method	28	Pakistan	SMS-based monitoring	Child immunization	SMS-based monitoring which involves use of SMS texts to ask the caregivers whether their children received the vaccine is an effective strategy to monitor immunization coverage following mass immunization campaigns.
2	Domek et al. 2016^44^	Randomized control trial (RCT)	321	Guatemala	SMS text reminders	Child immunization	No significant difference in those who received SMS and those who did not. However, high user satisfaction reported by those who received SMS
3	Bangure et al .2015^50^	RCT	304	Zimbabwe	SMS text reminders	Child immunization	Immunization coverage and adherence to immunization schedule was higher for those in the intervention group
4	Wakadha et al. 2013^48^	Intervention study	72	Kenya.	SMS reminder and Cash transfer	Child immunization	Cash incentive could be an effective strategy to increase immunization coverage and adherence
5	Domek et al. 2018^25^	RCT	1080	Guatemala	Mobile and SMS	Child immunization	Mobile ownership higher in urban areas, most women chosen by families for receiving reminders. In rural areas, landlines were used.
6	Nguyen et al. 2017^42^	Pre- and post-intervention		Vietnam	SMS texts	Child immunization	Significant increase in full immunization and measels; participants willing to pay for SMS reminders
7	Seth et al. 2018^52^	RCT	608	India	SMS messages	Child immunization	Delivery of automated mobile phone reminders increase infant immunization coverage
8	Kazi et al. 2018^28^	RCT	356	Pakistan	SMS Reminders	Child immunization	Intervention was successful however not a significant difference observed for 10 and 14 weeks schedule visit
9	Downs et al. 2019^29^	Intervention study	47	Senegal	Voice message	IYCF practice	Significant increase in consumption of fish and eggs
10	Flax et al. 2017^30^	Semi-structured exit interviews	195	Nigeria	Mobile phones, voice and text messages	Breastfeeding practices	Even sharing a messages with the microcredit group members once a week was associated with higher odds of breastfeeding
11	LeFevre et al. 2017^31^	Naturalist study design	22,237	Ghana	SMS text	Maternal health	Due to technological problems related with the platform used to deliver messages delay were observed in delivery of messages. Effectiveness of mHealth intervention was affected by functioning of the system
12	McBride et al. 2018^16^	Intervention study	961	Vietnam	SMS text	Maternal health	Increase in quality care among women from minority groups observed.
13	Modi et al. 2019^49^	RCT	6.493	India	Web-based technology	Maternal health	Improved coverage and quality of services in remote areas
14	Atnafu et al. 2017^47^	RCT	3.242	Ethiopia	SMS and mobile phone	Maternal health	Utilization of maternal health care services increased, however child immunization did not increase
15	Balakrishnan et al. 2016^27^	RCT	16,000	India	SMS and mobile phone	Maternal health	Service delivery increased both among marginalized and non-marginalized groups, and was higher in implementation block
16	Lund et al. 2012^56^	RCT	2550	Zanzibar	SMS and mobile phone voucher	Maternal health	Intervention increased coverage but rural women were left behind
17	Diese et al. 2018^32^	Mixed Method Study	190	Democratic Republic of Congo	SMS and mobile	Maternal health	The perception of households and local health service providers was positive regarding the use of mobile phone for monitoring of maternal and child health
18	Khan et al. 2018^33^	Qualitative study	24	Bangladesh	Mobile phones	Maternal health	Community health worker considered unreliable as per respondents and a higher willingness among all to receive information through mobile on IYCF
19	Tang et al. 2019^43^	Cross sectional Study	4494	Bangladesh	Mobile phones	Maternal health	Mobile phone users relative to nonmobile users have higher odds of using maternal health services
20	Hackett et al. 2019^34^	Cross-sectional study	14	Tanzania	Mobile phone	Maternal health	Positive perception and attitude of beneficiaries and community members towards use of smartphone application designed to improve data management, patient tracking and delivery of health messages during prenatal counseling visits with women clients
21	Murthy et al. 2019^35^	RCT	2016	India	Voice message	Maternal health	Significant improvement in infant care and maternal knowledge
22	Bangal et al. 2018^36^	RCT	400	India	Mobile phone and SMS	Maternal health	Higher number of antenatal care visits, institutional deliveries, postnatal checkup and lower perinatal mortality observed for intervention group
23	Coleman et al. 2020^37^	Intervention study	87	South Africa	SMS text	Maternal health	Health information text messages sent to women during pregnancy related with positive adherence to all first-year vaccinations for infants and four ANC visits
24	Domek et al. 2019^38^	RCT	720	Guatemala	SMS text reminders	Maternal health	Caregivers who were sent text message reminders were less delayed for their child's immunization visits and reported high user satisfaction when compared to caregivers of usual care group who did not get messages
25	Oladepo et al. 2021^39^	RCT	3440	Nigeria	SMS text	Maternal health	Messages increased awareness of immunization dates, assisted in timely completion
26	Mekonnen et al. 2021^26^	Cross-sectional study	456	Northwest Ethiopia	Use of mobile text message reminders	Maternal health	Majority of mothers have the intention to use text message reminders for child vaccination
27	Ekhaguere et al. 2019^40^	RCT	600	Nigeria	SMS reminders	Maternal health	Paired automated call and text reminders significantly improved immunization completion and timeliness
28	Dissieka et al. 2019^41^	RCT	798	Côte d’Ivoire	Voice or SMS reminders	Maternal health	Providing mothers with SMS reminder messages increased the proportion of child immunizations

ANC: antenatal care; IYCF: infant and young child feeding; mHealth: mobile health; SMS: short messaging service.

## Results

### Identification of studies

The search of literature yielded a total of 573 studies using PubMed, Cochrane Reviews, Google Scholar, and grey literature. After removing duplicates and studies not related to mHealth and MCH, 141 studies remained. Further, 93 research articles which were related to family planning and depression were excluded. Finally, a total of 43 studies were identified for full-text review of which 15 were systematic reviews and protocols. Out of the 28 studies, 15 studies were RCTs.^24,26,27,34,35,37,38,40–46^ The study design of the research articles which appeared during the search process were mostly observational, cross-sectional, and RCTs. We have classified the studies into four categories based on the outcomes for which the mHealth intervention was implemented: MCH care services, child immunization, nutrition services, and perceptions of stakeholders toward using technology for improving MCH outcomes.

### Study type

Of the 28 studies considered, 15 were RCTs.^24,26,27,34,35,37,38,40–46^ There is one study that measures the effectiveness of immunization coverage and timeliness of vaccination in pre- and post-intervention period. There are three qualitative studies, in which the variable of interest is the attitude and perception of the community members and community health workers on use of mobile communication for utilization and outcome of MCH care services.

Ten of the studies that have been identified are for the countries in the Africa continent such as Zimbabwe, Kenya, Senegal, Nigeria, Ghana, Ethiopia, Congo, Tanzania, Côte d’Ivoire, and South Africa. Five studies were identified for India, and two each for Pakistan and Bangladesh. Also, two studies were identified for Vietnam. In studies relating to both MCH, the target population is the caregiver of the child or pregnant and lactating women. The outcome variable for child health-related studies is the coverage of doses of vaccine at different time points. And, in case of maternal health, the outcomes for the studies are ANC, prenatal care (PNC), and institutional delivery. Two studies have documented the experience and attitude of the community health workers for the delivery of services and data management.

### Summary of studies

Our review of literature suggests that mobile phones can be used to achieve rapid improvement in utilization of MCH care services and outcomes. We observe that mHealth interventions are an effective strategy to quickly improve immunization coverage by setting reminders through use of SMS health messages. Similarly, we observe that the MCH outcomes were better among mobile phone users who received message and calls through the mHealth interventions when compared to nonmobile users. The salient findings based on the review are as follows.

#### MCH

Nine studies were identified on maternal health, of which three were RCTs, three were intervention studies, two qualitative, and one cross-sectional respectively. Most of these studies have documented increase in quality and service delivery among women who used mHealth-based interventions. In rural Ethiopia, SMS-based intervention was observed to be associated with improvement in maternal and child outcomes. More specifically, the rate of ANC delivered improved (from 19.01% to 28.27%), proportions of deliveries attended by skilled health workers increased and home delivery decreased in rural Ethiopia. Although mHealth interventions increase utilization of services, impact on certain indicators such as contraceptive use and immunization rates is still uncertain.^47^ In tribal dominated areas of Gujarat, use of ImTeCHO mobile- and web-based application as a job aid by government Accredited Social Health Activists (ASHAs) and PHC staff led to improvement in coverage and quality of MCH care services in hard-to-reach areas. Home visits by ASHAs during ANC period (adjusted effect size 15.7 [95% CI: 11.0, 20.4], *p* < 0.001) and postnatal period (adjusted effect size 6.4, [95% CI: 3.2, 9.6], *p* < 0.001) increased. Improvement was also reported in breastfeeding-related indicators. A study conducted in slums of Mumbai also reported that tailored mobile voice messages significantly improved infant care practices and maternal knowledge.

#### Child immunization

Six of the studies for increasing immunization coverage, using mHealth interventions, were RCTs. Five of these RCTs were conducted at health facilities. All the interventions involved delivery of SMS reminder to the caregivers regarding immunization schedule of the child just a few days prior to commencement of the immunization day. Bangure et al. reported that the immunization coverage at 6 weeks was 97% in the intervention group and 82% in the nonintervention group (*p* < 0.001) in Kadoma, Zimbabwe. Dissieka et al. in Côte d’Ivoire observed that infants in the intervention group were 2.85 (95% CI: 1.85–4.37), 2.80 (95% CI: 1.88–4.17), 2.68 (95% CI: 1.84- 3.91), and 4.52 (95% CI: 2.84–7.20) times more likely to receive pentavalent 1–3 and MMR/yellow fever doses. Domek et al. found that caregivers in the intervention in Guatemala were more likely to be present on the scheduled date when compared those in the control area. Similarly, Ekhaguere et al. report that infants in the intervention group were more likely to receive PENTA 3 (84% vs. 78%, RR 1.09, 95% CI 1.01 to 1.17; *p*  =  0.04), measles (73% vs. 65%, RR 1.13, 95% CI 1.02 to 1.26; *p*  =  0.02), and all scheduled immunizations collectively (57% vs. 47%, RR 1.13, 95% CI 1.02 to 1.26; *p*  =  0.01) within 1 week of the recommended date.

Kazi et al. also found that the immunization coverage in urban squatter settlement area of Karachi was consistently higher in the intervention group at the 6 weeks scheduled visit (76.0% vs. 71.3%, *p*  =  .36). Surprisingly, one study conducted by Domek et al. found nonstatistical difference in immunization coverage across intervention and control area. This could be because of high immunization rates across the region.

Only one study was located, which focused on the power of monetary incentives to modify the behavior of care giver, however, a survey in Vietnam indicates that parents are willing to pay for SMS reminders.^42,48^ SMS text messages are preferred when compared to interviews based on calls for monitoring.^23^

The findings from these studies indicate that willingness to receive messages was high among the caregivers. Most of the caregivers agreed that getting the reminder prior to the immunization schedule was helpful.^35,42^ Although the delivery of SMS reminders is the preferred approach, but voice messages might be a better option since there are wide disparities with respect to literacy rates and language might pose as a barrier.^17,31^ In addition, it is easier to track whether the caregiver heard the voice message or not. Moreover, poor network might lead to nondelivery of SMS reminders at times.^34^ Furthermore, some of the studies highlight barriers which might undermine the effectiveness of mHealth intervention such as distance from health facilities, inadequate stock of vaccine, low mobile phone ownership and poorly maintained immunization registries.

#### Nutrition services

Two studies were identified which evaluated the impact of mHealth interventions on infant and young child feeding (IYCF) practices. The focus of one of these interventions was on improving breastfeeding outcomes. The preferred mode to increase awareness about nutrition as per these studies is voice-based message. Following the delivery of voice messages to parents of children over a 4-week period in Senegal a significant increase in the number of children (6–23 months) that consumed fish (60% vs. 94%; *p*  =  .008) as measured by the 24-hour recall after the completion of the intervention was observed. We also found significantly higher frequency of egg (*p*  =  .026), fish (*p*  =  .004), and thick porridge (*p*  =  .002) consumption.^18^ In another study in Nigeria, sharing of text messages and voice messages among group members of micro-credit program was associated with higher odds of breastfeeding among women who met weekly.^19^ Electronic interventions have been found effective in promotion of breastfeeding.^24^ In a study in Bangladesh, mobile phones were identified as a valuable medium to deliver messages related to IYCF.^22^ The common challenge encountered during implementation is that participants at times do not provide accurate phone numbers. However, delivery of messages in groups attending weekly meetings as a part of micro-credit program seems an innovative approach to implement good practices related to IYCF which has the potential to create a spillover effect.

#### Perception of stakeholders toward using technology

In Democratic Republic of Congo, majority of the stakeholders believed that the use of mobile phones could lead to improvement in delivery of MCH care services. As per the respondents the best time to undertake these mHealth-based activities is the afternoon when women are generally alone and have more free time.^21^ Yet, another study evaluated the effectiveness of the Mobile Technology for Health (MOTECH) program in Ghana and reported that despite majority of women owning a phone less than 25% received the voice messages through the platform being used for the intervention.^20^ The reason for failure was observed to be missing phone-related data. Moreover, at the service provider level, such interventions could put more burden on the existing human resources leading to weak implementation. The intervention was able to benefit the most marginalized since voice messages were being delivered and it did not require women to be literate. Similarly, in Tanzania, community health worker in intervention areas were trained to use a smartphone application designed to improve data management, patient tracking and delivery of health messages during PNC counseling visits with women clients.^25^ Overall, the perception of using smartphones for delivery of quality messages was observed to be positive among pregnant women.

## Discussion

The emergence of mHealth programs and their incorporation into public health system of developing countries have indicated toward their potential to improve the delivery of health care solutions even in the remote areas.^6,49^ Our review of 28 studies have found mostly a favorable effect of mHealth intervention toward MCH especially for immunization.

Some of the key findings based on this review are as follows. First, even in the absence of any mHealth interventions, women who have a mobile phone are twice more likely to use maternal health services during pregnancy and deliver at health facilities.^43^ Clearly, targeted interventions can increase coverage of MCH care services. Technology does not discriminate, and if the system is working efficiently then it has the power to enhance equity in service coverage among both marginalized and non-marginalized groups.^27^ However, the interventions work well when women have a mobile phone for their own use. Therefore, there is a need to understand the trend and pattern in mobile ownership across various geographical levels.

Second, experience of other countries indicates that SMS is an effective tool to improve the immunization coverage and to modify the health seeking behavior.^11,44,50^ In fact, conditional money transfer is an important tool to leverage the power of mobile payment system to incentivize the households to get the children vaccinated.^11^ Such strategies might be highly effective in not only resource constrained settings but also in regions where baseline coverage of MCH is high, and a lot of efforts are required to achieve marginal improvement in coverage.^51^ Although some of the studies are based on a small sample size but there is no denying the fact that mHealth-based intervention can be effective to increase immunization coverage.^45,52^ Overall, the attitude and perception of both health workers and those seeking health care services was favorable toward adopting mobile phones.

Coming to the challenges, findings from some of the studies indicate that health workers who are of low socio-economic status might not have complete information about the messages which are being sent through the mHealth platform. This might defeat the entire purpose of the mHealth intervention since ultimately, the care seekers interact with health workers and might risk getting unreliable information.^33^ Therefore, training the workers and capacity building of the health workers is an equally important requirement for mHealth interventions to be successful. It is worth noting that developing countries have a shortage of human resources in the health system. Introduction of mHealth intervention can increase the workload of the healthcare workers in the field and affect the service delivery. Therefore, caution is required before aggressively scaling up such interventions so that delivery of services is not affected. Furthermore, the unavailability of digital infrastructure such network and poor functioning technology platforms can lead to implementation failure.

Furthermore, there is a close link between gender equality, dietary diversity, and malnutrition.^53^ Mobile ownership is a sign of women empowerment. The power of mobile phone can be leveraged to improve dietary diversity among women during pregnancy as well as child health by improving breastfeeding practices.^54^ However, further studies are required to understand the distribution of mobile ownership among women and to assess the effectiveness of such studies at a large scale. Lastly, technology can also lead to increase in inequalities in access to healthcare by empowering those who are able to use it. For instance, despite the tremendous growth in access to mobile phones and internet-based services, mobile ownership among woman remain low. Particularly, women who have a lower socio-economic status have limited access to mobile phones.^46,55^ Also, women in rural areas are less likely to have phone and might be unable to benefit from the mHealth interventions.^56^ Clearly, mHealth interventions can aggravate socio-economic inequalities.

There are certain limitations to this study. Although these studies document the effectiveness of the mHealth interventions, little has been mentioned about the problems faced during early stage of implementation of the intervention. Initially, the adoption might be difficult since technology supported platform might not work efficiently or the distribution of mobile ownership might be skewed. Also, sample size for these studies is small, therefore, the results cannot be generalized. The settings in which the research was conducted can influence the results. However, the review does draw the conclusion from the experience of several countries which lend credibility to the results that the interventions work. There are regions where social norms and lack of infrastructure can lead to failure in implementation of mHealth interventions.

## Conclusion

There are a number of objectives for which mHealth interventions can be used such as data collection, interaction with health care providers, creating awareness about best practices for improving MCH, modifying behavior of the caregivers, and ensuring adherence through reminders for follow-up. This review concludes that mHealth interventions can be instrumental in improving the access to maternal health services. In addition, incentive linked compliance through text messaging is another approach which could ensure higher immunization coverage. Such gains achieved through mHealth interventions could help in achieving the targets related to maternal and child mortality.

Though most of the studies identified in our review are based on a small sample size and confined to specific sites; however, overall, the findings are consistent enough to support the effectiveness of the mHealth interventions across intervention groups. In addition, RCT across small regions can provide evidence about “what works” which paves the way for scaling up the interventions across neighboring area. To evaluate the success of the m-health programs, a greater understanding of the barriers as well as constraints is required since the existing literature does not unravel the reason for attrition during the course of the intervention. Further research is warranted from the perspective of implementation science which could help in integrating mHealth interventions with existing health-based interventions to reap gains at a larger scale.

## Supplemental Material

sj-docx-1-dhj-10.1177_20552076221143236 - Supplemental material for Effect of mobile health interventions in increasing utilization of Maternal and Child Health care services in developing countries: A scoping review Click here for additional data file. Supplemental material, sj-docx-1-dhj-10.1177_20552076221143236 for Effect of mobile health interventions in increasing utilization of Maternal and Child Health care services in developing countries: A scoping review by Ramachandran Venkataramanan, S.V. Subramanian, Mohannad Alajlani and Theodoros N Arvanitis in Digital Health
