# Supplementary material for: Effect of mobile health interventions in increasing utilization of Maternal and Child Health care services in developing countries: A scoping review
Source: Digit Health. 2022 Dec 13;8:20552076221143236. doi: 10.1177/20552076221143236 (PMC9756363; doi:10.1177/20552076221143236)
Supplement: sj-docx-1-dhj-10.1177_20552076221143236 - Supplemental material for Effect of mobile health interventions in increasing utilization of Maternal and Child Health care services in developing countries: A scoping review [file sj-docx-1-dhj-10.1177_20552076221143236.docx]

Supplementary material

PubMed Full Search Strategy

"mHealth or mobile health" AND ("maternal health services" OR "child health" or "pregnancy" or

"immunization" or "vaccination") AND “Developing countries”
